# Online decision support for persons having a genetic predisposition to cancer and their partners during reproductive decision‐making

**DOI:** 10.1002/jgc4.1056

**Published:** 2018-12-21

**Authors:** Kelly Reumkens, Marly H. E. Tummers, Joyce J. G. Gietel‐Habets, Sander M. J. van Kuijk, Cora M. Aalfs, Christi J. van Asperen, Margreet G. E. M. Ausems, Margriet Collée, Charlotte J. Dommering, C. Marleen Kets, Lizet E. van der Kolk, Jan C. Oosterwijk, Vivianne C. G. Tjan‐Heijnen, Trudy van der Weijden, Christine E. M. de Die‐Smulders, Liesbeth A. D. M. van Osch

**Affiliations:** ^1^ Department of Clinical Genetics Maastricht University Medical Centre+ Maastricht The Netherlands; ^2^ GROW School for Oncology and Developmental Biology Maastricht University Medical Centre+ Maastricht The Netherlands; ^3^ Department of Clinical Epidemiology and Medical Technology Assessment Maastricht University Medical Centre+ Maastricht The Netherlands; ^4^ Department of Clinical Genetics Academic Medical Centre Amsterdam The Netherlands; ^5^ Department of Clinical Genetics Leiden University Medical Center Leiden The Netherlands; ^6^ Division of Biomedical Genetics, Department of Genetics University Medical Center Utrecht Utrecht The Netherlands; ^7^ Department of Clinical Genetics Erasmus University Medical Center Rotterdam The Netherlands; ^8^ Department of Clinical Genetics VU University Hospital Amsterdam The Netherlands; ^9^ Department of Human Genetics Radboud University Medical Center Nijmegen Nijmegen The Netherlands; ^10^ Family Cancer Clinic Netherlands Cancer Institute Amsterdam The Netherlands; ^11^ Department of Genetics Groningen University Medical Center, University of Groningen Groningen The Netherlands; ^12^ Department of Medical Oncology Maastricht University Medical Centre+ Maastricht The Netherlands; ^13^ Department of Family Medicine, Faculty of Health, Medicine and Life Sciences, School CAPHRI Maastricht University Maastricht The Netherlands; ^14^ Department of Health Promotion, Faculty of Health, Medicine and Life Sciences, School CAPHRI Maastricht University Maastricht The Netherlands

**Keywords:** counseling, decision aid, genetics, hereditary cancer, informed decision‐making, oncology, patient participation, reproductive decision‐making

## Abstract

A nationwide pretest–posttest study was conducted in all clinical genetic centres in the Netherlands, to evaluate the effects of an online decision aid to support persons who have a genetic predisposition to cancer and their partners in making an informed decision regarding reproductive options. Main outcomes (decisional conflict, knowledge, realistic expectations, level of deliberation, and decision self‐efficacy) were measured before use (T0), immediately after use (T1), and at 2 weeks (T2) after use of the decision aid. Paired sample *t* tests were used to compute differences between the first and subsequent measurements. T0–T1 and T0–T2 comparisons indicate a significant reduction in mean decisional conflict scores with stronger effects for participants with high baseline decisional conflict. Furthermore, use of the decision aid resulted in increased knowledge levels and improved realistic expectations. Level of deliberation only increased for participants with lower baseline levels of deliberation. Decision self‐efficacy increased for those with low baseline scores, whereas those with high baseline scores showed a reduction at T2. It can be concluded that use of the decision aid resulted in several positive outcomes indicative of informed decision‐making. The decision aid is an appropriate and highly appreciated tool to be used in addition to reproductive counseling.

## 1 INTRODUCTION

Most hereditary cancer syndromes follow an autosomal‐dominant inheritance pattern, implying that there is a 50% risk of transmitting a pathogenic variant to offspring, with a high risk of a future malignancy as a consequence. For the relatively frequent breast cancer gene mutations in *BRCA1* or *BRCA2*, this implies risks of 27%–57% and 6%–40% of developing breast respectively ovarian cancer by the age of 70 (Brohet et al., 2014; Chen & Parmigiani, 2007). Persons having a genetic predisposition to cancer and their partners have to make fundamental decisions about future reproduction and face difficult challenges (Dekeuwer & Bateman, 2013; Derks‐Smeets et al., 2014; Donnelly et al., 2013; van Asperen et al., 2002). Couples have three options to fulfill their wish for a child that is genetically related to both parents. The first option is natural conception without genetic testing, implying acceptance or taking the risk of passing on the pathogenic variant. Furthermore, there are two options for having a genetically related child to both parents without a pathogenic variant. The first option is natural conception with prenatal diagnosis (PND), offering the choice to terminate the pregnancy if the fetus has the pathogenic variant (de Die‐Smulders, de Wert, Liebaers, Tibben, & Evers‐Kiebooms, 2013). The second option is preimplantation genetic diagnosis (PGD). PGD offers the option to obtain embryos by in vitro fertilization (IVF) and screen them for the familial pathogenic variant. Only embryos without the pathogenic variant are transferred into the uterus (de Die‐Smulders et al., 2013). Levels of awareness for PND (61%) and PGD (66%) are similar, and couples consider PGD (80%) to be more acceptable for hereditary cancer compared to PND (26%) (Gietel‐Habets et al., 2017).

Couples may experience difficulties with reproductive decision‐making (Dekeuwer & Bateman, 2013; Dommering et al., 2010; Ormondroyd et al., 2012), and it was reported that for some, even years later, the impact of reproductive decision‐making still had an influence on their lives at a daily basis (Derks‐Smeets et al., 2014). In deliberating the options, couples consider personal values and (dis)advantages of the options, such as physical (e.g., burden of PGD treatment), psychological (e.g., loss of sense of romance), social (e.g., elimination of the pathogenic variant in family line), ethical (e.g., moral duty to protect the child), and practical considerations (e.g., reimbursement of treatment) (Derks‐Smeets et al., 2014). Which reproductive option suits them best, should ideally be decided in an informed decision‐making process by an educated and empowered couple, supported by a dedicated health care provider. In order to promote informed reproductive decision‐making, the use of decision aids can be effective (Derks‐Smeets et al., 2014; Juraskova et al., 2014; O'Connor & Jacobsen, 2003; Quinn et al., 2010; Stacey et al., 2017). The present study is part of a larger study on the development and implementation of an online decision aid, developed in accordance with the International Patient Decision Aids Standards (Reumkens, Oudheusden, et al., 2018; Reumkens, Tummers, et al., 2018; Volk, Llewellyn‐Thomas, Stacey, & Elwyn, 2013). In this study, we report on the effects of the decision aid evaluated in a nationwide pretest‐posttest study in all clinical genetic centres in the Netherlands.

## 2 METHODS

### 2.1 Participants and recruitment

Health care providers (e.g., clinical geneticists) of all Clinical Genetics Departments in the Netherlands recruited eligible couples during or after oncogenetic consultations from January 2017 to January 2018. Couples were eligible for participation if one partner had a pathogenic variant predisposing for autosomal dominant hereditary cancer, for which PND and PGD are available in the Netherlands. These hereditary cancers include, but are not limited to carriers and partners of carriers of the following types of hereditary cancer: hereditary breast and ovarian cancer (HBOC), hereditary colon cancer (e.g., familial adenomatous polyposis (FAP), hereditary non‐polyposis colorectal cancer (HNPCC/Lynch Syndrome), Peutz–Jeghers syndrome, multiple endocrine neoplasia (MEN1/2), retinoblastoma, Von Hippel Lindau disease, Li–Fraumeni syndrome, familial atypical multiple mole/melanoma syndrome (FAMMM). Furthermore, couples needed to have the intention to have children within the next 5 years, and had not yet made a definitive decision regarding their preferred reproductive option. Both partners had to be 18 years or older and both partners needed to have sufficient knowledge of the Dutch language.

### 2.2 Procedures

Eligible couples were provided with an information brochure including a link to an online registration page. After registration, both partners received an informed consent form by e‐mail. After providing online informed consent, both partners were individually directed to an online (baseline) questionnaire (T0). Questionnaires were completed separately by both partners. Subsequently, they received a personal login code for the decision aid. It was allowed to use the decision aid together. Duration of use and page visits were monitored. Immediately after use of the decision aid, participants were directed to the second questionnaire (T1). Two weeks after baseline, participants were asked by e‐mail to complete a third questionnaire (T2). A reminder was sent to participants who did not complete the T1 questionnaire within 1 day, or the T2 questionnaire within 7 days. An incentive of 15 euros in vouchers was provided after completion of all questionnaires. This study was approved by the medical ethics committee of Maastricht UMC+ (METC 14‐5‐089).

### 2.3 Content of the decision aid

An extensive explanation of the developmental process and the specific content of the decision aid are provided elsewhere (Reumkens, Tummers, et al., 2018). Overall, the decision aid contained:

1. Information about the risk of transmitting the pathogenic variant to offspring and couples’ options to have genetically related children.
2. Treatment burden of reproductive options and the chances of different outcomes (e.g., risk of miscarriage after PND) presented in multiple suitable formats using text and videos (e.g., verbal, and population diagrams) (Reumkens, Oudheusden, et al., 2018; Trevena et al., 2013).
3. A comparative summary table of important features of each option.
4. Value clarification exercises (VCE) (Fagerlin et al., 2013). A total of 18 statements represents values and motives considered important for reproductive decision‐making (Derks‐Smeets et al., 2014).
5. By linking login codes, a combined overview of both partners’ responses on the VCE was provided.
6. A question prompt sheet, providing examples of questions and requests for additional information and space for own questions.
7. Information regarding the scientific resources used to underpin the decision aids content, the development team, funding resources, and contact information.

### 2.4 Instrumentation

Gender, age, educational level, carrier status, disease type, number of children and couples’ planning for having children were assessed at T0. Less than primary education, primary and lower secondary education were considered as low education levels. Upper secondary and post‐secondary non‐tertiary education were considered as middle education levels. Tertiary education was considered as a high education level. At T0 and T2, couples were also asked if they already had a consultation with a healthcare provider. The main subject of this consultation (1 = solely focusing on the reproductive options, 2 = focusing on the consequences of having the pathogenic variant, the reproductive options concerned only a small part) and the profession of the healthcare provider were assessed.

The primary outcome measure, that is, participants’ level of ***decisional conflict*** (at T0, T1, T2), was assessed by the Decisional Conflict Scale (O’Connor, 1995a). The questionnaire contained 16 items (Cronbach's *α* = 0.82). Three items (*α* = 0.90) were used to assess values of uncertainty about the decision, three items (*α* = 0.84) assessed feelings of being informed, three items (*α* = 0.90) assessed personal beliefs regarding the reproductive options, three items (*α* = 0.63) assessed feelings of being supported in making a reproductive decision, and four items (*α* = 0.82) assessed the feeling of having made an effective decision. Each item was scored on a 5‐point Likert scale ranging from 0 (strongly agree) to 4 (strongly disagree). Total scores ranged from 0 (no decisional conflict) to 100 (extremely high decisional conflict) (O'Connor, 1995a). As the items in the effective decision subscale could not be completed by couples who did not have a preferred reproductive option in mind, a combined score was also calculated for the four other subscales. These 12 items were summed, divided by 12, and multiplied by 25. Total scores ranged from 0 (no decisional conflict) to 100 (extremely high decisional conflict).

Participants’ current ***knowledge of the three reproductive options*** (at T0, T1, T2) was assessed by 15 items (Gietel‐Habets et al., 2017). Three questions measured participants’ knowledge of natural conception without genetic testing (e.g., “When opting for natural conception, there is a 50% risk of transmitting the pathogenic variant to offspring”; 1 = correct, 2 = incorrect, 3 = not sure), five questions measured knowledge of PND (e.g., “PND takes place during pregnancy”) and seven questions measured knowledge of PGD (e.g., “IVF is necessary to perform PGD”). One point was provided to each correctly answered question, with a maximum score of 15.

Participants’ ***decision self‐efficacy*** (at T0, T1, T2) was assessed by the Decision Self‐Efficacy Scale (Bunn & O’Connor, 1996). The questionnaire contained 11 items (Cronbach's *α* = 0.84), each scored on a 5‐point Likert scale ranging from 0 (not at all confident) to 4 (very confident). Total scores ranged from 0 (extremely) to 100 (extremely high) (Bunn & O’Connor, 1996).

***Realistic expectations*** regarding the reproductive options (T0, T1, T2) were assessed by three questions (i.e., “What is the extra risk of miscarriage due to PND?”, “What is the chance of pregnancy after one IVF treatment with PGD?”, “What is the risk of complications with PGD?”). These questions contained 8 to 11 answer options. One point was provided to each correctly answered question, with a maximum score of 3 (O'Connor, 1995b).

***Level of deliberation*** (T0, T1, T2) was measured by the Deliberation Scale (Van den Berg, Timmermans, Kate, Vugt, & Wal, 2006). The questionnaire contained six items (Cronbach's *α* = 0.90), each scored on a 5‐point Likert scale ranging from 1 (totally disagree) to 5 (totally agree). Total scores ranged from 6 (low level) to 30 (high level).

***Evaluative items*** (T1). Participants were asked to give an overall appreciation score for the decision aid at a scale from 1–10 and to indicate in open‐ended questions positive and negative features and possibilities for improvements. Furthermore, 10 items (e.g., perceived efficiency and active trust) were used to assess user perceptions (Crutzen et al., 2014) including two items of the system usability scale (SUS) (Brooke, 1996). Each item was scored on a 5‐point Likert scale ranging from 0 (totally disagree) to 4 (totally agree).

Lastly, participants’ ***preparation for decision‐making*** (T1) was measured by the Preparation for Decision Making Scale (Graham & O’Connor, 1995). The questionnaire contained 10 items (Cronbach's α = 0.92), each scored on a 5‐point Likert scale ranging from 1 (totally not) to 5 (a lot). Total scores ranged from 0 (low level) to 100 (high level) (Graham & O’Connor, 1995).

### 2.5 Data analysis

Data from the baseline characteristics were analyzed by means of descriptive statistics. Cohen's d was used to report effect sizes; Cronbach's alpha was computed to assess reliability. Furthermore, an intra‐couple correlation test was performed before evaluating effects. We compared two models to test for intra‐couple correlation regarding the main outcome (decisional conflict); one linear mixed‐effects model in which clustering within participants over time and within couples was corrected for, and one model without correction for clustering within couples. Both models yielded similar results, and a likelihood‐ratio test showed that correction for the clustering of observations within couples did not lead to a better model fit (likelihood ratio = 0.00, *p* = 1.000). Therefore, all participants were analyzed as independent from each other and therefore we chose to report the simpler model without correction for clustering and used the paired sample *t* test to compute differences between the first and subsequent measurements. For in‐depth analyses, a median split was performed for all main outcome measures. Analyses were performed using IBM spss version 23 and r version 3.3.3. *p*‐values of <0.05 were considered to indicate statistical significance.

## 3 RESULTS

### 3.1 Baseline characteristics

A total of 140 participants visited the registration page, of which 133 provided informed consent. T0 was completed by 115 participants (86.5%) and 110 participants actually visited the decision aid (82.7%). 102 participants completed T1 (76.7%) and 86 participants completed T2 (64.7%). 80.4% of the participants filled out the T1 questionnaire immediately after visiting the decision aid. T2 was on average filled out 17.7 days after T0 (*SD *= 10.05). The mean time spent using the decision aid was 27 min (range 5–95 min) and participants viewed a mean of 15 of 36 pages. Table 1 shows an overview of baseline characteristics. The average age of males (*M* = 31.6, *SD *= 3.6) was slightly higher compared to females (*M* = 29.2, *SD *= 2.9). Most participants were highly educated (57.4%). The most frequently reported hereditary cancer syndrome was HBOC (85.2%).The majority of the participants (89%) already had a consultation in which the reproductive options were discussed. The consultation, mostly with clinical geneticists, focused mainly on the consequences of having the pathogenic variant (58.9%). In 41.1% of the participants, the reproductive options had been the main topic. The majority of the participants had heard of PND (73.0%) and PGD (89.6%) before participation in this study and most of them had also received information: on PND: 59.1%, on PGD: 69.6%. A little over half of the participants (51.4%) had a preferred reproductive option in mind at baseline.

**Table 1:** Baseline characteristics (*N* = 115)

	*N*	Percentage
Gender		
Male	51	44.3
Female	64	55.7
Age (years)		
Male	31.6 (*SD* 3.6)	
Female	29.2 (*SD* 2.9)	
Education		
Low	11	9.6
Middle	38	33.0
High	66	57.4
Carrier status		
Male carrier	36	31.3
Female carrier	79	68.7
Syndrome		
HBOC	98	85.2
Lynch syndrome	8	7.0
FAP	2	1.7
Li–Fraumeni syndrome	2	1.7
Melanoma syndrome	1	0.9
Hereditary diffuse gastric cancer syndrome	2	1.7
Hereditary leiomyomatosis and renal cell cancer	2	1.7
Children		
Yes	20	17.4
No	95	82.6
Planning to have children		
Trying to conceive now	11	9.6
Within 2 years	70	60.9
Within 5 years	28	24.3
Not sure yet	4	3.5
Otherwise	2	1.7

Note

FAP: Familial Adenomatous Polyposis; HBOC: Hereditary breast and ovarian cancer.

### 3.2 Effects of the decision aid

As shown in Table 2, total mean *decisional conflict* scores (range 0–100) for all five subscales significantly decreased from 25.30 at baseline, to 18.06 at T1 (Effect Size (ES) = 0.73) and 17.22 at T2 (ES = 0.51). Total mean decisional conflict scores (range 0–100) excluding the effective decision subscale, significantly decreased from 35.54 at baseline, to 25.33 at T1 (ES = 0.78) and 26.31 at T2 (ES = 0.44). In‐depth analyses (Table 3) indicated that participants with high baseline decisional conflict scores (≥33), excluding the effective decision subscale, had a significant reduction in total scores from baseline (*M* = 51.35) to T1 (*M* = 34.46; ES = 1.29) and T2 (*M* = 34.72; ES = 0.80) whereas participants with low baseline decisional conflict scores (<33) only showed a significant reduction in total scores at T1 (ES = 0.43).

**Table 2:** Effects of use of the decision aid on main outcome measures

	T0 (baseline)	T1 (immediately after use of the decision aid)	T2 (2 weeks after baseline)	T0–T1			T0–T2		
	Means (SD)	Means (SD)	Means (SD)	*T*	*p*	*ES*	*T*	*p*	*ES*
Decisional conflict									
Total score (0–100)^a^	25.30 (11.61)	18.06 (11.60)	17.22 (13.46)	5.78	<0.001	0.73	3.65	0.001	0.51
Total score (excl. effective decision; 0–100)	35.54 (19.03)	25.33 (15.74)	26.31 (19.50)	7.88	<0.001	0.78	4.11	<0.001	0.44
Uncertainty	44.14 (28.00)	35.89 (25.81)	34.12 (24.15)	5.10	<0.001	0.51	4.58	<0.001	0.50
Informed	31.60 (20.25)	18.07 (13.28)	20.10 (16.48)	7.24	<0.001	0.72	4.51	<0.001	0.49
Values clarity	34.32 (22.40)	23.60 (17.99)	22.25 (18.56)	5.79	<0.001	0.58	5.21	<0.001	0.57
Support	32.10 (19.49)	23.76 (16.64)	22.16 (16.74)	6.07	<0.001	0.60	4.93	<0.001	0.53
Effective^a^ decision	22.22 (16.72)	15.77 (15.10)	12.98 (14.21)	3.68	<0.001	0.46	3.48	0.001	0.48
Knowledge									
Total score (0–15)	9.28 (2.76)	13.16 (1.85)	12.63 (1.90)	−13.89	<0.001	−1.37	−10.28	<0.001	−1.11
Natural conception (0–3)	2.29 (0.64)	2.66 (0.57)	2.81 (0.65)	−5.58	<0.001	−0.55	−5.81	<0.001	−0.62
PND (0–5)	2.14 (1.29)	3.92 (1.10)	3.73 (1.47)	−12.64	<0.001	−1.25	−8.14	<0.001	−0.86
PGD (0–7)	4.85 (1.54)	6.58 (0.83)	6.42 (0.95)	−11.03	<0.001	−1.09	−8.69	<0.001	−0.92
Realistic expectations (0–3)	0.72 (0.71)	1.63 (1.12)	1.08 (0.96)	−9.08	<0.001	−0.85	−3.92	<0.001	−0.37
Level of deliberation (6–30)	23.23 (4.47)	23.90 (3.70)	24.07 (3.42)	−1.39	0.168	−0.16	−1.28	0.204	−0.16
Decision self‐efficacy (0–100)	77.23 (12.20)	79.43 (15.39)	79.81 (14.21)	−1.57	0.119	−0.16	−2.11	0.037	−0.23

a *N *= 63 for T0–T1; *N* = 52 for T0–T2.

**Table 3:** In‐depth analyses for main outcome measures based on median split baseline scores

	T0 (baseline)	T1 (immediately after use of the decision aid)	T2 (2 weeks after baseline)	T0–T1			T0–T2		
	Means (SD)	Means (SD)	Means (SD)	*T*	*p*	*ES*	*T*	*p*	*ES*
Decisional conflict (0–100)^a^									
Low baseline (<33)	21.23 (9.77)	17.06 (11.13)	18.79 (16.00)	3.16	0.003	0.43	0.82	0.419	0.12
High baseline (≥33)	51.35 (13.36)	34.46 (15.10)	34.72 (19.78)	8.92	<0.001	1.29	5.16	<0.001	0.80
Knowledge (0–15)									
Low baseline (≤10)	6.93 (2.12)	12.70 (2.04)	12.11 (1.94)	−14.04	<0.001	−2.07	−11.11	<0.001	−1.83
High baseline (>10)	11.21 (1.36)	13.54 (1.61)	13.02 (1.80)	−10.38	<0.001	−1.39	−6.57	<0.001	−0.94
Realistic expectations (0–3)									
Low baseline (≤1)	0.54 (0.50)	1.52 (1.11)	0.98 (0.89)	−9.32	<0.001	−0.92	−4.85	<0.001	−0.48
High baseline (≥2)	2.15 (0.38)	2.46 (0.88)	1.85 (1.14)	−1.17	0.264	−0.32	0.94	0.367	0.26
Level of deliberation (6–30)									
Low baseline (≤24)	19.44 (4.06)	22.19 (3.36)	22.27 (3.46)	−3.22	0.003	−0.57	−2.68	0.013	−0.53
High baseline (>24)	27.22 (2.03)	26.48 (2.99)	26.39 (3.14)	1.43	0.166	0.27	1.30	0.208	0.27
Decision self‐efficacy (0–100)									
Low baseline (≤75)	67.00 (7.42)	73.07 (15.16)	75.78 (13.59)	−3.20	0.003	−0.47	−6.59	<0.001	−1.03
High baseline (>75)	88.18 (6.98)	86.11 (13.95)	82.45 (14.60)	0.92	0.361	0.14	2.22	0.033	0.37

a Decisional conflict scale excluding effective decision subscale.

As shown in Table 2, the mean level of *knowledge* (range 0–15) significantly increased from 9.28 at baseline, to 13.16 at T1 (ES = −1.37) and 12.63 at T2 (ES = −1.11). In‐depth analyses (Table 3) indicated that knowledge scores significantly increased for both participants with high (>10) and low (≤10) baseline knowledge levels.

As shown in Table 2, *Realistic expectations* (range 0–3) significantly increased from 0.72 at baseline, to 1.63 at T1 (ES = −0.85) and 1.08 at T2 (ES = −0.37). In‐depth analyses (Table 3) showed that realistic expectations were significantly increased at T1 and T2 for participants with low (≤1) baseline levels.

As shown in Table 2, with a mean score of 23.23 (range 6–30), the level of *deliberation* was relatively high at baseline and did not show an overall increase over time. However, in‐depth analyses (Table 3) indicated that participants with lower baseline levels of deliberation (≤24) showed a significant increase over time from 19.44 at baseline, to 22.19 at T1 (ES = −0.57) and 22.27 at T2 (ES = −0.53). No effect was found for participants with higher baseline levels of deliberation (>24).

As shown in Table 2, participants’ *decision self‐efficacy* (range 0–100) did not significantly increase from baseline (77.23) to T1 (79.43; ES = −0.16). From baseline to T2, decision self‐efficacy significantly increased (*M* = 79.81; ES = −0.23). In‐depth analyses (Table 3) indicated that decision self‐efficacy of participants with low baseline scores (≤75) significantly increased from 67.00 at baseline to 73.07 at T1 (ES = −0.47) and 75.78 at T2 (ES = −1.03), whereas those with high baseline scores (>75) showed a significant reduction in self‐efficacy at T2 (ES = 0.37).

### 3.3 Depth of use of the decision aid

As shown in Table 4, both users with low engagement (≤15 pages) and users with high engagement (>15 pages) showed decreased decisional conflict scores, increased knowledge levels, and increased realistic expectations at T1 and T2 (all *p*'s < 0.05; decisional conflict in high engagement group: *p* = 0.05). Only users with high engagement showed increased levels of deliberation and increased decisional self‐efficacy at T1 and T2 (all *p*'s < 0.05).

**Table 4:** Effects of the decision aid related to depth of use

	T0 (baseline)	T1 (immediately after use of the decision aid)	T2 (2 weeks after baseline)	T0–T1			T0–T2		
	Means (SD)	Means (SD)	Means (SD)	*T*	*p*	*ES*	*T*	*p*	*ES*
Decisional conflict (0–100)^a^									
Low engagement^b^	35.75 (21.12)	27.08 (15.83)	25.57 (16.99)	4.48	<0.001	0.63	3.93	<0.001	0.59
High engagement^c^	35.55 (16.95)	23.58 (15.85)	27.79 (22.35)	6.58	<0.001	0.96	2.02	0.050	0.32
Knowledge (0–15)									
Low engagement	9.22 (2.97)	12.37 (2.03)	11.95 (1.93)	−7.59	<0.001	−1.06	−5.64	<0.001	−0.85
High engagement	9.34 (2.64)	14.00 (1.14)	13.39 (1.62)	−12.94	<0.001	−1.89	−8.96	<0.001	−1.45
Realistic expectations (0–3)									
Low engagement	0.64 (0.76)	1.45 (1.14)	1.08 (1.03)	−5.49	<0.001	−0.75	−3.25	0.002	−0.45
High engagement	0.84 (0.65)	2.08 (0.90)	1.24 (0.87)	−9.81	<0.001	−1.39	−2.92	0.005	−0.41
Level of deliberation (6–30)									
Low engagement	23.63 (4.72)	22.87 (4.33)	23.51 (3.99)	1.13	0.268	0.18	0.20	0.842	0.03
High engagement	22.58 (4.23)	24.78 (2.64)	24.67 (2.72)	−3.43	0.002	−0.57	−2.31	0.028	−0.42
Decision self‐efficacy (0–100)									
Low engagement	77.68 (13.42)	77.64 (16.85)	77.38 (15.19)	0.02	0.985	0.00	0.04	0.968	0.01
High engagement	76.89 (11.44)	81.19 (14.21)	82.00 (12.96)	−2.67	0.010	−0.39	−3.65	0.001	−0.60

Decisional conflict scale excluding effective decision subscale. ^b^≤15 pages. ^c^>15 pages.

### 3.4 Evaluation of the acceptability

The mean score on the Preparation for Decision Making Scale (range 0–100) was 62.3 (*SD *= 19.6). A majority (82.2%) thought it was easy to find information in the decision aid (*M* = 3.24, *SD *= 0.81), found the various functions well integrated (84.2%, *M* = 3.05, *SD *= 0.73), the information offered consistent (90.1%, *M* = 3.43, *SD *= 0.70), and relevant (82.6%, *M* = 3.41, *SD *= 0.64). Furthermore, participants found the decision aid easy to use (90.1%, *M* = 3.22, *SD *= 0.74) and trusted the offered information (94.1%, *M* = 3.40, *SD *= 0.63). A majority (80.2%) indicated that their awareness regarding the available options increased (*M* = 2.97, *SD *= 0.88), 93.5% thought that it would be useful to develop the decision aid also for other hereditary diseases (*M* = 3.51, *SD *= 0.66), 94.1% would recommend the decision aid to others (*M* = 3.51, *SD *= 0.64) and 80.2% (*M* = 3.05, *SD *= 0.89) would use the decision aid again in the future.

Participants graded the decision aid on a scale of 1–10 with a mean of 8.2 (*SD *= 0.94). The avoidance of medical or technical terms was appreciated and the provided information was clear, neutral (i.e., not guiding) and comprehensible. Particularly, the value clarification exercises and informational videos were appreciated, but the inclusion of narrative stories, translation into the English language and making it better compatible for use on mobile devices were frequently mentioned improvements.

## 4 DISCUSSION

To our knowledge, this is the first study to report on the effects of a decision aid to support persons having a genetic predisposition to cancer and their partners in decision‐making regarding their reproductive options. Overall immediate (T0–T1) and sustained (T0–T2) effects were found for decisional conflict, knowledge, and realistic expectations, only sustained effects were found for decisional self‐efficacy. No main effects were demonstrated for the level of deliberation. However, analyses on depth of use of the decision aid showed that users with high engagement showed a significant effect on all outcome measures. This indicates that using the decision aid to its full extent positively influences all main outcome measures. Furthermore, in‐depth analyses showed both immediate and sustained effects in increasing deliberation among those with lower baseline levels of deliberation. This indicates that the decision aid is capable of encouraging deliberation among couples who are in the early stages of decision‐making (Elwyn & Miron‐Shatz, 2010). Furthermore, in‐depth analyses showed stronger effects for participants with lower baseline levels of realistic expectations, self‐efficacy, and high levels of decisional conflict, further corroborating the conclusion that the decision aid particularly supports couples with higher needs for reproductive decision support.

A notable finding is the small but significant reduction in decisional self‐efficacy scores at T2 for participants with high baseline scores, indicating that use of the decision aid introduced some uncertainty among those who felt confident in their decision‐making ability at baseline. A solid knowledge base is regarded as a prerequisite for informed decision‐making (Van den Berg et al., 2006). Post‐hoc analyses indicated that baseline knowledge levels were identical for participants with low and high baseline scores of decision self‐efficacy (*M* = 9.21 respectively *M* = 9.20), suggesting that the expressed confidence in decision‐making was not based on adequate knowledge levels. As the decision aid had such strong effects on knowledge of reproductive options, the information provided in the decision aid may have resulted in the identification of possible misconceptions or knowledge gaps, and possibly a further realization of the complexity of the decision among those with high baseline decisional self‐efficacy. This finding furthermore emphasizes the importance of embedding the decision aid in a counseling process with adequate follow‐up counseling for couples who are still in need of professional support after viewing the decision aid.

### 4.1 Study limitations

The use of a pretest‐posttest design restricts the internal validity, as maturation and history effects as well as effects due to repeated testing cannot be controlled for. Although the execution of measurements immediately before and after the use of the decision aid minimizes the likelihood of bias, possible interference of other factors, such as use of other information sources or different exposure to impactful counseling, cannot be excluded. Furthermore, the majority of the participants did not use the complete decision aid. This could be due to the length and amount of information in the decision aid. Further investigation of possible consequences of abbreviating the decision aid on the effectiveness of the decision aid is therefore recommended. Lastly, as urgency of child wish is not standardly registered in all clinical genetic centres and hospital regulations prohibited the distribution of non‐participating individuals, we cannot provide an estimate of the number of eligible couples and we were unable to calculate a response rate.

### 4.2 Research recommendations

The majority of the participants in this study were highly educated (57%). Although this is in line with general characteristics of oncogenetic counselees (Giessen van der, 2017), this number is notably high compared to the numbers in the general Dutch population (30%) (CBS, 2016). This further exposes the need for research on measures to improve referral of patients with a lower educational background. Furthermore, as the reproductive decision is often not implemented within several months after reproductive counseling or after reviewing the decision aid, a long‐term follow‐up to measure decision adherence (e.g., 18 months after reviewing the decision aid) would be useful.

### 4.3 Practice implications

Use of the decision aid resulted in several positive outcomes indicative of informed decision‐making which may lessen the negative psychological impact of decision‐making on couples’ daily life and well‐being. The decision aid is an appropriate and highly appreciated tool to be used in addition to reproductive counseling. In‐depth analyses showed that the couples who are in the highest need of reproductive decision support are those who are the most supported by the decision aid which increases the overall impact of the decision aid. Currently, we are conducting an explorative implementation study to clarify optimal timing of providing the decision aid and how to incorporate the decision aid in daily practice. To further increase the impact of the decision aid, the content of the tool will be adapted to other hereditary conditions. Supporting Information Data [Link], [Link], [Link].

## 5 CONCLUSION

The current findings indicate that the decision aid can be effective in supporting persons having a genetic predisposition to cancer and their partners in making an informed decision regarding reproductive options. Further research is needed to indicate prolonged effects on informed decision‐making and informed choice.

## ACKNOWLEDGEMENTS

I confirm that the work was conducted to fulfill a degree requirement or as part of training.

## 6 COMPLIANCE WITH ETHICAL STANDARDS

All procedures performed in this study were in accordance with the ethical standards of the medical ethics committee of Maastricht University Medical Centre and have been performed in accordance with the ethical standards as laid down in the 1964 Declaration of Helsinki and its later amendments.

### Conflict of interest

The authors declare that they have no conflict of interest.

### Human studies and informed consent

Informed consent was obtained from all individual participants included in this study.

### Animal studies

No animal studies were carried out by the authors for this article.

## 7 AUTHORSHIP CONTRIBUTIONS

K.R., M.H.E.T., L.A.D.M.v.O., and C.E.M.d.D.‐S have made substantial contributions to the conception and design of the study. All the authors except for S.M.J.v.K, V.C.G.T‐H, T.v.d.W and L.A.D.M.v.O made substantial contributions to recruitment of participants. K.R., M.H.E.T, C.E.M.d.D.‐S and L.A.D.M.v.O made substantial contributions to analysis and interpretation of data. All the authors have made substantial contributions to revising the article and final approval of the version to be published.
