# Supplementary material for: Online decision support for persons having a genetic predisposition to cancer and their partners during reproductive decision‐making
Source: J Genet Couns. 2018 Dec 21;28(3):533–42. doi: 10.1002/jgc4.1056 (PMC7380023; doi:10.1002/jgc4.1056)
Supplement: Supplementary file 2 [file JGC4-28-533-s002.docx]

**Posttest Survey (T2)**

**1. Child wish**0 Trying to conceive now
0 Child wish within two years
0 child wish within five years
0 Not sure yet
0 Otherwise, namely…………………..

**2. Did you already had a consultation with a health care provider in which the reproductive options were discussed?**0 Yes
0 No

**3. If yes, what was the main subject of this conversation?**0 This conversation was focussing on the available reproductive options
0 This conversation was mainly focussing on the consequences of having the pathogenic variant and the reproductive options were only a small part of the conversation

**4. The conversation was with:**0 Clinical Geneticist
0 Genetic counsellors
0 Gynaecologist
0 Oncologist
0 Psychologist
0 Otherwise, namely….

**5. Do you have a reproductive option in mind?**0 I did not yet considered the reproductive options
0 I did not yet considered the reproductive options, but I am interested in doing so
0 I am currently considering the reproductive options
0 I almost decided regarding my preferred reproductive option
0 I decided regarding my preferred reproductive option, but I am willing to reconsider
0 I decided regarding my preferred reproductive option and its unlikely that I will change my mind

**Knowledge questionnaire True-false-not sure**

1. When opting for natural conception, there is a 50% risk of transmitting the
   pathogenic variant to offspring
2. When opting for natural conception, besides standard procedures, there will be no extra examinations performed during pregnancy
3. When opting for natural conception, during delivery it is already clear whether your child has the pathogenic variant.
4. Prenatal diagnosis takes place during pregnancy
5. When opting for prenatal diagnosis , you and your partner can naturally conceive
6. Results of prenatal diagnosis will always follow within one week
7. Prenatal diagnosis is possible from 6 weeks of pregnancy upon
8. Prenatal diagnosis is possible in most of the medical centers in the Netherlands
9. In vitro fertilization (IVF) is necessary to perform PGD
10. PGD is possible in every hospital in the Netherlands
11. For PGD, cooperation of family members is a prerequisite
12. Hormone-use by the woman is necessary for a PGD treatment
13. PGD takes place before the woman is pregnant
14. A PGD treatment takes at least 6 months
15. In the Netherlands, a women’s maximum age for PGD is 45

**Realistic expectations questionnaire**

**1. What is the extra risk of miscarriage due to PND?**
0 100 out of 100 women will have a miscarriage due to prenatal testing
0 81-99 out of 100 women will have a miscarriage due to prenatal testing
0 61 – 80 pout of 100 women will have a miscarriage due to prenatal testing
0 41-60 out of 100 women will have a miscarriage due to prenatal testing
0 21-40 out of 100 women will have a miscarriage due to prenatal testing
0 11-20 out of 100 women will have a miscarriage due to prenatal testing
0 6-10 out of 100 women will have a miscarriage due to prenatal testing
0 1-5 out of 100 women will have a miscarriage due to prenatal testing
0 Less than 1 out of 100 women will have a miscarriage due to prenatal testing
0 there is no risk of a miscarriage due to prenatal testing
0 I don’t know

**2. What is the chance of pregnancy after one IVF treatment with PGD?**0 0-5 out of 100 women will be pregnant
0 10-15 out of 100 women will be pregnant
0 20-25 out of 100 women will be pregnant
0 30-40 out of 100 women will be pregnant
0 50-70 out of 100 women will be pregnant
0 80-99 out of 100 women will be pregnant
0 100 out of 100 women will be pregnant
0 I don’t know

**3. What is the risk of complications with PGD?**0 100 out of 100 women will have complications with PGD
0 81-99 out of 100 women will have complications with PGD
0 61-80 out of 100 women will have complications with PGD
0 41-60 out of 100 women will have complications with PGD
0 21-40 out of 100 women will have complications with PGD
0 11-20 out of 100 women will have complications with PGD
0 6-10 out of 100 women will have complications with PGD
0 1-5 out of 100 women will have complications with PGD
0 Less than 1 out of 100 women will have complications with PGD
0 0 out of 100 women will have complications with PGD
0 I don’t know

**Decision Self-Efficacy Scale** *5-point Likert scale ranging from 0 (not at all confident) to 4 (very confident)***I feel confident that I can:**1. Get the facts about the reproductive options available to me
2. Get the facts about the benefits of each reproductive option
3. Get the facts about the risks and side effects of each reproductive option
4. Understand the information enough to be able to make a reproductive choice
5. Ask questions without feeling dumb
6. Express my concerns about each reproductive option
7. Ask for advice regarding each reproductive option
8. Figure out the reproductive option that best suits me
9. Handle unwanted pressure from others in making my reproductive choice
10. Let the clinic team know what’s best for me
11. Delay my reproductive decision if I feel I need more time

**Deliberation Scale** *5-point Likert scale ranging from 1 (totally disagree) to 5 (totally agree)*
1. I have tried to mentally visualize the reproductive options
2. I have imagined how I would feel if I did not choose this reproductive option
3. I have imagined how I would feel if I did choose this reproductive option
4. I have tried to think through the consequences of not choosing this reproductive option
5. I have tried to think through the consequences of choosing this reproductive option
6. I have made a mental list of the pros and cons

**Reproductive decision**
**1. Did you already made a reproductive decision?**
0 Yes
0 No

**Decisional Conflict Scale**
*5-point Likert scale ranging from 0 (strongly agree) to 4 (strongly disagree)*1. I know which reproductive options are available to me
2. I know the benefits of each reproductive option
3. I know the risks and side effects of each reproductive option
4. I am clear about which benefits matter most to me
5. I am clear about which risks and side effects matter most
6. I am clear about which is more important to me (the benefits or the risks and side effects)
7. I have enough support from others to make a reproductive choice
8. I am choosing without pressure from others
9. I have enough advice to make a reproductive choice
10. I am clear about the best reproductive choice for me
11. I feel sure about what to choose
12. The decision regarding reproductive options is easy for me to make
13. I feel I have made an informed reproductive choice
14. My decision shows what is important to me
15. I expect to stick with my reproductive decision
16. I am satisfied with my reproductive decision
